# Discovering microproteins: making the most of ribosome profiling data

**DOI:** 10.1080/15476286.2023.2279845

**Published:** 2023-11-27

**Authors:** Sonia Chothani, Lena Ho, Sebastian Schafer, Owen Rackham

**Affiliations:** a Program in Cardiovascular and Metabolic Disorders, Duke-National University of Singapore, Singapore; b School of Biological Sciences, University of Southampton, Southampton, UK; c The Alan Turing Institute, The British Library, London, UK

**Keywords:** Ribo-seq, ribosome profiling, smorfs, Seps, RNA translation

## Abstract

Building a reference set of protein-coding open reading frames (ORFs) has revolutionized biological process discovery and understanding. Traditionally, gene models have been confirmed using cDNA sequencing and encoded translated regions inferred using sequence-based detection of start and stop combinations longer than 100 amino-acids to prevent false positives. This has led to small ORFs (smORFs) and their encoded proteins left un-annotated. Ribo-seq allows deciphering translated regions from untranslated irrespective of the length. In this review, we describe the power of Ribo-seq data in detection of smORFs while discussing the major challenge posed by data-quality, -depth and -sparseness in identifying the start and end of smORF translation. In particular, we outline smORF cataloguing efforts in humans and the large differences that have arisen due to variation in data, methods and assumptions. Although current versions of smORF reference sets can already be used as a powerful tool for hypothesis generation, we recommend that future editions should consider these data limitations and adopt unified processing for the community to establish a canonical catalogue of translated smORFs.

## Small open reading frames: missed key players in biology

**Figure 1.**
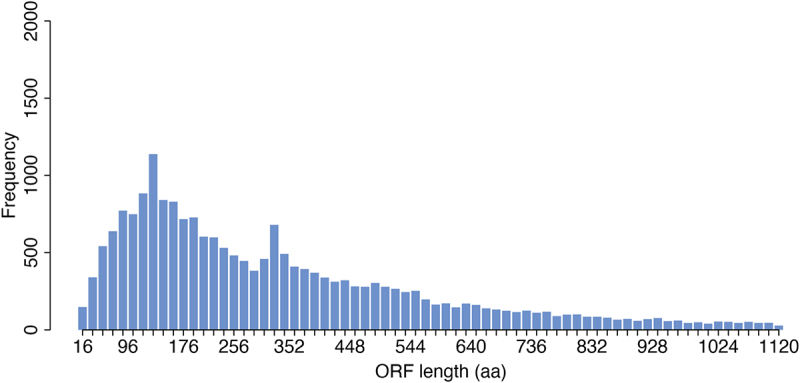
Length distribution of known protein-coding ORFs. Barplot showing frequency of ORFs annotated in Ensembl (hg38) according to their length. Frequency of ORFs longer than 1200 amino-acids are not shown.

To date, there are ~ 22,000 genes annotated in Ensembl [1] that contain an open reading frame (ORF, known ORFs) and which are considered ‘protein coding’. However, the bioinformatic process by which these annotations were made included assumptions about ORF length. This was done in order to account for the fact that start and stop codons appear at random in the genome and as such there are many millions of genomic loci that have ORF-like characteristics [2] (i.e. they are between a start and a stop codon), but only a fraction of these are translated. As such, in the past, to ensure reliable prediction of ORFs a threshold of ~ 100 amino acids has often been used to ensure the validity of predicted ORFs. Not including this assumption would have resulted in the annotation of many millions of possible small ORFs (smORFs), most of which would have been false positives. However, as a consequence we have systematically missed those ORFs that are shorter than 100 amino acids, despite there being no biological reason for their exclusion (see Figure 1).

Evidence is increasingly accumulating that we may have underestimated the prevalence of smORFs. Genes previously annotated as non-coding (e.g. long-non coding RNAs or lncRNA) have been found to be frequently associated with mono- and polysomal complexes [3], suggesting many more may be translated. Indeed, dozens of smORF encoded peptides (SEPs) have been found in lncRNAs playing a role in a diverse range of biological functions (referred to as novel unannotated ORFs or nuORFs). Early studies showed that translation of smORFs located upstream of known ORFs (referred to as upstream ORFs or uORFs) could play a cis-regulatory effect (both negative and positive) [4] on the host ORF, and more recently uORFs have also been shown to encode functional peptides [5–7]. Equally, small open reading frames have been identified downstream of ORFs (known as dORFs). Taken together, these discoveries raise the question of to what extent might a significant subset of the millions of possible small ORFs (smORFs) in the ‘dark matter’ of the genome that were previously disregarded are actually translated?

A step-change in smORF identification came with the introduction of Ribosome profiling (or also called Ribo-seq) [8,9]. This technology can generate a snapshot of ribosome locations across the transcriptome with single nucleotide resolution. For the first time, Ribo-seq provided experimental evidence to estimate global translation levels in vivo [10–12]. Importantly, Ribo-seq was able to demonstrate prevalent binding of ribosomes to RNA outside of the known coding regions. However, there have been disagreements in the field as to what extent of the previously annotated non-coding regions are translated [13,14]. As a result, the challenge that remains is how to accurately distinguish active translation from ribosome occupancy. Specifically, we outline the data, methods and assumptions used in development of reference sets of translated smORFs in humans, the current versions of such sets and the overlap across them. We highlight the relative sparseness of data despite the large number of studies, and following from this, argue for combining multiple Ribo-seq datasets to improve signal-to-noise ratios for smORF identification. We also caution against simply aggregating predictions into consensus smORF reference sets without standardizing the smORF annotation pipeline. Moving forward, we recommend pooling Ribo-seq data and adopting unified processing standards to establish a high-quality, consensus smORF catalogue for the research community. It should be noted that computational tools to detect translated smORFs, classification of smORFs and exemplar SEPs have been reviewed in detail previously [15–17] and as such will not be covered in detail here.

## The 3-nt periodicity observed using Ribo-seq has revolutionized smORF detection

**Figure 2.**
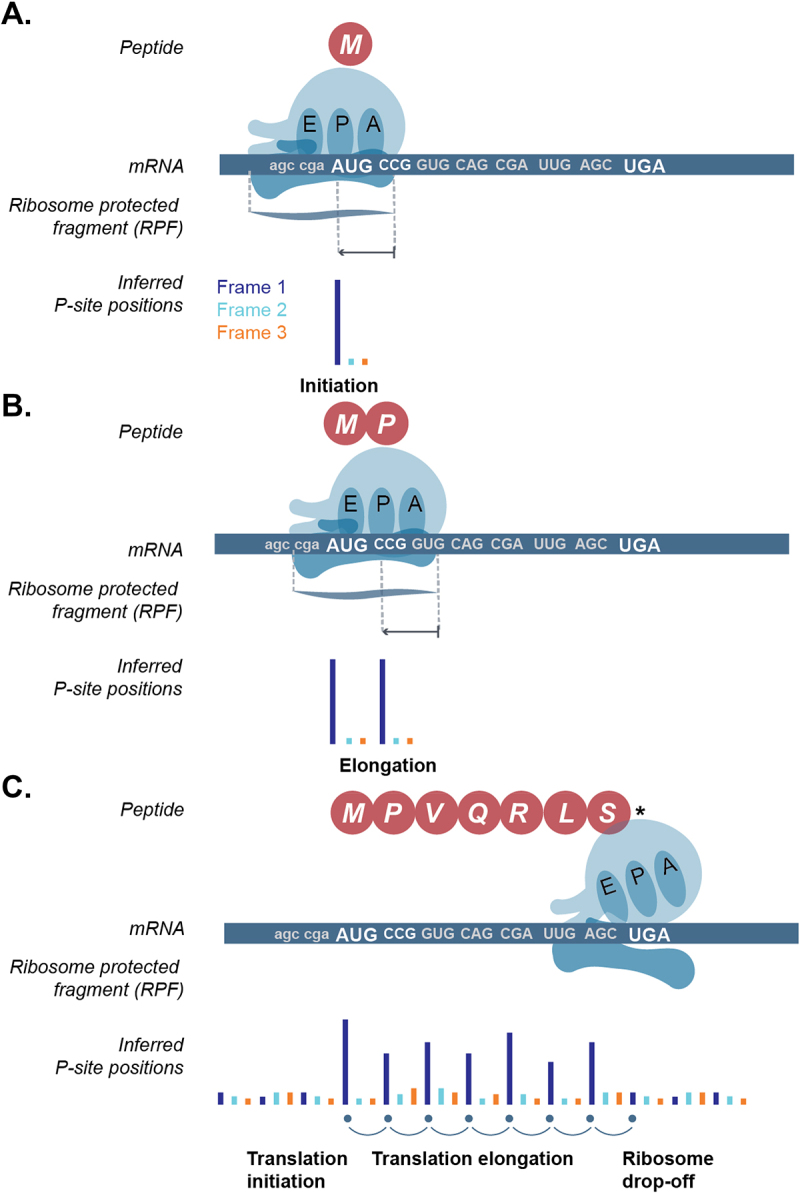
Three-nucleotide periodicity profile of ribosome footprints during active translation of an open reading frame (ORF). A. Schematic showing translation initiation on the canonical start codon (AUG, methionine) of the ORF, B. translation elongation i.e. translation of the subsequent codons (only one codon shown) of the ORF after initiation, and, C. translation termination with ribosome drop-off or disassembly at the stop-codon (UGA here). E-site: exit site, P-site: peptidyl-site, A-site: aminoacyl site. Sequenced ribosome protected fragment (RPF) can be used to infer the position of the P-site and subsequently the codon that is being translated. Inferred P-site position is coloured based on the ORF frame. Frame 1: dark blue, frame 2: light blue, frame 3: orange.

Many RNA-ribosome interactions are unrelated to translation, and thus polysome profiling is not ideal to discover translated ORFs [18]. Detection of smORFs using Ribo-seq is potentially more accurate and has been a very active field of research since its discovery. Initial studies have shown that ribosome footprints can be found in the known ORFs but also within regions that had previously been annotated as ‘non-coding’. However, these initial studies did not take advantage of the single-nucleotide resolution of Ribo-seq data [19]. Active translation of mRNA leads to ribosome footprints with inferred P-sites on the first nucleotide of every codon (or every three base-pairs)-, thus forming a three-nucleotide periodic signal (or periodicity, see Figure 2) observed at a nucleotide resolution. In contrast, a random ribosomal occurrence (i.e. one that is unrelated to mRNA translation) leads to footprints in all nucleotides uniformly. Periodicity observed by combining the ribosome protected fragments (RPFs) around the start and stop of known ORFs has been routinely used to examine the data quality [12,20–23]. Subsequently, scanning the transcriptome for regions with 3-nt periodicity has been at the heart of smORF detection [24].

## Current catalogues present a wide-range and inconsistent sets of smORFs

**Figure 3.**
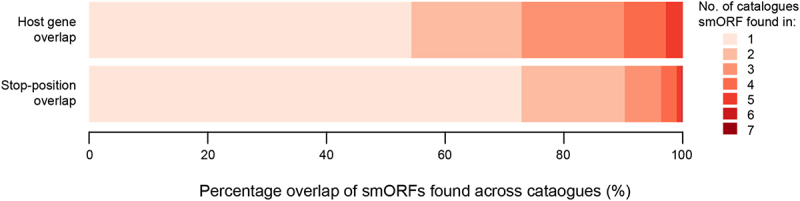
Overlap of smORFs identified across different published catalogues. Stacked barplot showing percentage of smORFs found in various catalogues. A smORF is considered as found in multiple catalogues if it shares the host gene ID (from five catalogues) or shares the same stop-site position (from seven catalogues). Catalogues which did not have host gene symbol information in the downloads file were omitted [28,32] for the host-gene overlap.

Numerous catalogues listing smORFs using existing Ribo-seq data either to detect or to validate translation have been generated but disparities in data and methods used to identify actively translated smORFs can lead to large differences in the annotation of translated smORFs. Across the seven catalogues [25–32] described (discussed in detail below) in this review, we found that less than 50% of the smORFs could be found in at least two catalogues when using the host gene ID as a reference. This overlap was reduced to ~ 27% for two catalogues and less than 10% for three catalogues when using the exact stop-site position to test for repeated identification (see Figure 3). Although repeated identification in independent catalogues do not necessarily provide unequivocal evidence for the veracity of smORF, and likewise a smORF found only in one catalogue may still be bona fide, the question remains as to how large is the consensus and what should be considered a reference set for future studies. The lack of a consensus reference set such as those for known ORFs (i.e. Uniprot [33], Refseq [34] and Ensembl [1]) creates challenges for researchers trying to understand the function and clinical utility of smORFs.

In an ideal-scenario, translated smORFs would be identified based on continuous 3-nt periodicity from their start-to-end (as in Figure 2) using Ribo-seq as evidence of their translation. In reality, currently available individual sample data are very sparse with uneven coverage across ORFs. The computational methods being developed to detect smORFs have numerous approaches for accounting for this sparseness, with varying levels of stringency. As a result, large differences in smORF detection can occur simply by fluctuations in data quality and smORF detection algorithms, something that is often overlooked. As the field moves towards constructing a consensus set of smORFs these aspects must be considered and accounted for. For this reason, this review will focus on the challenges faced by the community and best practices that the field should consider adopting to reach the goal of a canonical smORF catalogue.

## Are the underlying data of high-resolution or not?

**Figure 4.**
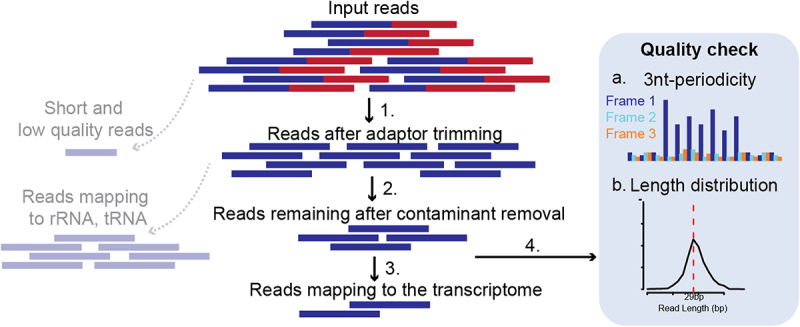
Pre-processing steps and quality control of sequenced Ribo-seq reads. 1. Sequencing adaptors are trimmed from input reads. Short and low-quality reads are discarded. 2. Contaminant sequences such as reads mapping to ribosomal RNA (rRNA) or transfer RNA (tRNA) are discarded. 3. Remaining reads are mapped to the transcriptome. 4. Quality control steps are carried out such as 3-nt periodicity (a) and length distribution (b).

Ribo-seq data provide an unprecedented resolution of translation but the available data is very sparse both due to technical and biological limitations. The sample library prep requires high input material and has several optimization steps that can potentially generate distortions as described previously [24]. Thus, sequenced Ribo-seq reads undergo several pre-processing steps including strict quality control and only selected high-quality usable reads are used to infer P-site positions for smORF detection (see Figure 4). Short and low-quality sequenced reads are discarded after trimming sequencing adaptors followed by mapping of remaining reads to a database of contaminant sequences including ribosomal RNA (rRNA) and transfer RNA (tRNA). Such contaminant sequences are very prevalent in Ribo-seq data, making this step very detrimental to the usable read depth. This typically results in as low as only ~ 10% [35] of the sequenced reads to be usable. From the remaining reads about ~ 20–80% are reported as uniquely mapped to the transcriptome in various studies, considering the short read length and varying data quality [36]. Apart from these pre-processing steps, QC such as length distribution and 3-nt periodicity is also tested. The ribosomal footprint during active translation is expected to have a fixed read length range depending on the digestion conditions [37], cellular stress [38] or elongation inhibitor [39]. In a typical Ribo-seq experiment, when cycloheximide is used as the elongation inhibitor, the most prevalent footprints should be ~ 29 nt long in eukaryotic cytosolic ribosomes [8]. Failure to observe these footprints with high 3-nt periodicity in coding sequences may suggest that the data is not reliable for identifying actively translated mRNA. Despite this, many datasets, generated under normal conditions, using cycloheximide, fail to achieve these periodic footprints (see Supp. Figure 1). Overall, a high number of input reads and optimization is required to obtain high-depth and -quality Ribo-seq data that can be used to detect smORF translation accurately and such data are limited.

**Figure 5.**
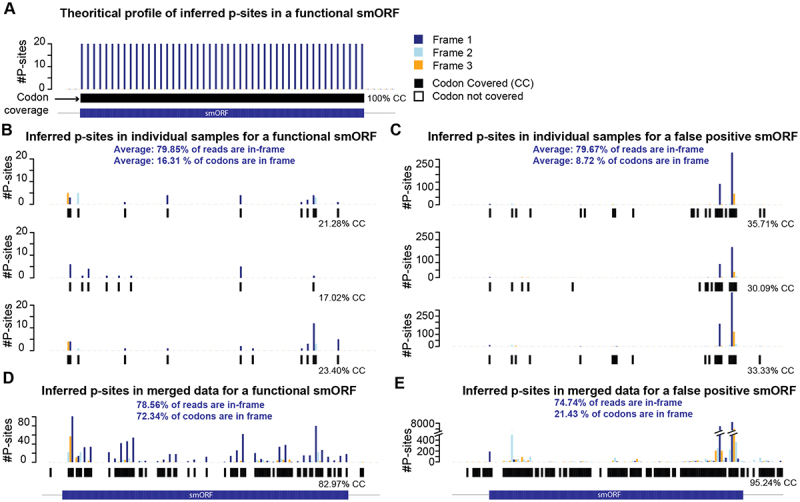
Evidence of translation using Ribo-seq inferred P-sites is sparse across ORFs. A. Barplot showing theoretical distribution of inferred P-site positions (#P-sites) and codon-coverage based on Ribo-seq reads in a given smORF. B. Barplot showing inferred P-site positions and codon coverage across the smORF region using individual samples from hepatocytes [31] for a known and functionally validated smORF SEHBP [44] (B), and a false positive smORF located at chromosome 2: 70,087,581 –70,087,706 (C). D-E. barplot showing inferred P-site positions and codon coverage for smORF shown in B (D), and C (E) using merged high-quality Ribo-seq data [31]. SmORF region is marked in dark blue. P-sites in frame 1: dark blue, frame 2: light blue and frame 3: orange.

A low usable read-depth of Ribo-seq leads to low coverage across all codons in ORFs. For instance, in the human genome, there are more than 12 million codons [1] within known proteins. Using a single sample of say 20–30 million sequenced reads typically yields roughly less than, 5–10 million uniquely mapped reads (after QC filters as described above). Therefore, this barely provides one read per codon or P-site location. In reality, this number would be even lower as the coverage is confounded by several factors. First, coverage depends on expression levels and ORFs that are lowly expressed would be more difficult to detect [40]. Second, Ribo-seq typically has non-uniform coverage across codons leading to no-information available for many codons and thus making it impossible to determine if those regions were translated. For instance, the availability of tRNAs can influence the speed of translation by stalled or rapid translation of certain codons, thus leading to higher or lower RPFs mapping to those codons, respectively [41]. Previous studies have shown that there is non-uniform coverage across the length of ORFs [42,43]. Theoretically, with sufficient depth and population size, 100% codons would have Ribo-seq read coverage in the translating frame, providing a clear picture of smORF translation (Figure 5A). In reality, various biological and technical biases lead to non-uniform coverage and limited usable read depth. This leads to absence of coverage in several codons. A study showed that in individual Ribo-seq samples, 84% codons are covered only for the top 10 expressed genes while for top 1000 expressed genes, only 36% of the codons are covered [45]. Here, we show an example of a validated uORF encoded protein (SEHBP) [44], using individual sample data only ~ 20% of codons are covered (>1 inferred P-site read) providing no evidence of translation for more than 80% of codons (see Figure 5B). For another example, which is a predicted smORF with no known function, likely a false-positive smORF, also shows only ~ 33% codons covered (Figure 5C). In both the examples, it becomes impossible to confirm the translation of the full length of the smORF to differentiate it from artefacts or false positives as well as accurately define its coordinates. Overall, currently available Ribo-seq samples do not have uniform coverage at the nucleotide-resolution throughout the smORF, thus making it difficult to confirm smORF translation and its coordinates accurately. As such, Ribo-seq can illuminate new translated regions of the genome, but challenges and questions remain as to how best to achieve this.

## Is the ORF actively translated?

Several metrics and tools have been developed to detect actively translated smORFs keeping in mind the sparseness of Ribo-seq data. Since there is not enough depth at the nucleotide-resolution, translation of a smORF is often tested by a summarized view of reads mapped to the smORF. For instance, if a smORF is translated, it would be expected to have RPFs of length distribution similar to the distribution within known coding sequences in the given Ribo-seq experiment and the Fragment length organization similarity score (or FLOSS) [14] uses this information to determine a smORFs coding potential. Similarly, if a smORF is translated it is expected to have more reads mapped within the ORF as compared to after its stop codon. The Ribosome release score (or RRS) [13] quantifies the ratio of the reads in the putative smORF and the following 3’UTR normalized to the lengths of the regions and mRNA read depth to test for a smORF’s coding potential. TOC classifier [46] uses a random forest classifier that models on scores such as RRS, FLOSS, inside-out (metric to test nucleotides covered by Ribo-seq inside the ORF and outside the ORF) and translational efficiency.

The above metrics look for features of translation holistically, but they do not confirm the continuous translation of the smORF. The ideal scenario for smORF detection would be observing periodic RPFs over every codon in the smORF. However, because of the shallowness in a typical Ribo-seq library, observing such 3nt-periodicity over every codon of an ORF is rare. For this reason, many of the published methods use strategies to overcome this, for example, combining the observations across codons or alleviating the shallowness. For example, Ribotaper [47] uses a multitaper strategy followed by a Fourier transform as a way to detect periodic signals across all codons. Spectre [48] uses a sliding window approach and RibORF [49] bins the ORF into regions based on the available read coverage within the ORF. RiboWave [50] uses a chi-square test to test for in-frame P-site enrichment in the ORF in comparison to the flanking regions. RiboNT [51] compares data in the translating frame with the two other frames using student t-tests to infer presence of periodicity in smORFs. ORFscore [52] compares the ORF’s RPF distribution in each frame with an equally sized uniform distribution using a chi-square test and additionally quantifies % of in-frame positions with reads. Alternatively, in order to alleviate shallow usable data, PRICE [53] attempts to rescue reads such as multi-mappers that would otherwise be discarded. Another tool, RiboHMM [54], assigns modified emission probabilities for base positions with missing data according to the current state of the HMM (e.g. TES, 5’UTS, TIS). Rb-Bp [55] applies LOWESS smoothing for each frame to account for sparse or spiky nature of Ribo-seq data. These tools allow the detection of actively translated smORFs with 3-nt periodicity throughout the length of the smORF, although care must be taken while interpreting the results as they largely depend on data depth and quality.

## Where does the smORF start?

The 3-nt periodicity confirms the translating frame and the first encountered stop in this translating frame determines the end of the smORF encoded protein. Determining the start is relatively more difficult. Alternative start-sites and non-canonical start-codons [56] increase the complexity of start-site determination and subsequently can exponentially increase the number of possible isoforms for a given smORF. The sparseness in Ribo-seq data makes it difficult to decipher the most-used start of the smORF and for simplicity, several studies apply prior assumptions such as limiting possible start to only AUG or selecting the most 5’ AUG (or longest) as the start-site [49,52,57,58]. Some studies use Ribo-seq data coverage to determine the most used start-site such as SmProt [30], which uses the highest ‘–framebest’ score from RiboTISH [59] tool to select the isoform with the best coverage. Shorter isoforms have a bias towards full coverage, thus another study used the 5’ most start-site which maintains uniform coverage of periodicity [31]. Variants of Ribo-seq protocol have been also developed for enrichment of translation initiation (TI) sites using drugs that preferentially inhibit translation initiation only such as harringtonine [9], lactimidomycin [60] and lactimidomycin followed by puromycin [61,62] which is a translation inhibitor that effectively depletes elongating ribosomes. TIS data analysis suggests that the majority of ribosomes initiate translation at cognate AUG codons, followed by near cognate start codons CUG, GUG with ~ 50% initiating at non-AUG start-codons [9,61,62]. Therefore, is critical to consider non-canonical initiation sites when defining smORF start-sites. Computational tools to combine TI-seq and Ribo-seq data from the same biological samples have been developed such as ORF-Rater [63], which uses a regression fit against an expected profile of start- and stop-signals. TISCA [64], which combines translation complex sequencing (TCP-seq) to determine the 40S ribosomal subunit decreasing point along with global TI-seq to more accurately determine initiation sites. RiboTISH [59], detects initiation sites and also quantifies differential initiation site usage across conditions using TI-seq data. Similar to the issue with detecting translation, the start-site determination is also largely dependent on data-depth and quality. Different tools deploy varying prior assumptions and methods to determine the start of a given smORF and care must be taken in interpreting results and combining them.

## Pooling data to define a reference set of smORFs

**Figure 6.**
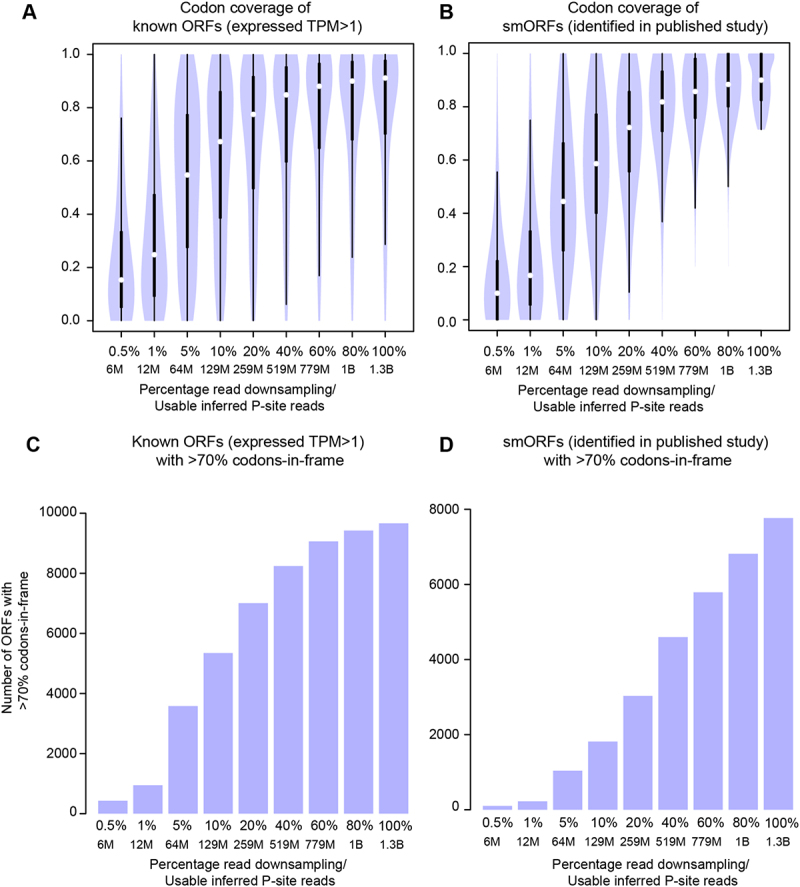
Increase in codon coverage and codons-in-frame within ORFs using pooled Ribo-seq data. A-B. Violin plot showing codon coverage (>1 Ribo-seq read with inferred P-site in the given codon) found with varying usable read depth for known ORFs from Ensembl (A) and smORFs identified in a previous study (B). C-D. Barplots showing number of ORFs with more than 70% codons-in-frame using data with varying usable read depth for known ORFs from Ensembl (C) and smORFs identified in a previous study [31] (D). Usable read depth is the number of inferred P-sites obtained after filtering low-quality sequencing reads, adaptor trimming, removal of contaminant sequences such as rRNA and selecting only uniquely mapped reads for the read lengths that show 3-nt periodicity for annotated ORFs (between 28–30 bp). Known ORFs were filtered for expression (TPM > 1 in at least one sample).

The ultimate goal of smORF detection is to identify potential peptides encoded by genomic regions that could be incorporated into our knowledge base of known proteins. As smORFs were previously excluded only for technical practicality, with new technologies providing high-resolution for translation of smORFs, their reference set development efforts should be treated no different from known ORFs. Historically, gene models were defined using cDNA data which transitioned to using RNA-seq for improved accuracy of 3’UTR boundaries and splice junctions. With the aim to obtain a reference set for a given species, these Ensembl gene models were built based on a pooled dataset with RNA-seq reads merged across tissue-types. Individual tissue dataset gene-models were only used for further refining [65]. In stark contrast, currently, most studies defining smORF sets use single-samples to detect smORF coordinates. This causes two problems: First, considering the sparseness of Ribo-seq data there is not enough evidence to distinguish translation from noise in individual sample data, increasing the false positives (Figure 5B,C). Second, this has led to multiple cell- and tissue-type reference sets instead of a common reference set for a given species. To address the first issue, a study combined three replicates of the same tissue in arabidopsis for smORF detection increasing the codon coverage to 90% [66]. Similarly, technical or biological replicates have been merged in few other studies [30,32]. To address both the issues, in humans, recently a study pooled reads from all published and newly generated high-quality and QC-passed human Ribo-seq from 11 primary human cells and tissues. This led to 1.3 billion inferred P-sites which covered ~ 80% codons across the genome [31]. Trips-Viz [67] also uses aggregated data from multiple studies to improve detection and then uses several features (such as number of codons in regions of interest with higher in-frame reads as compared to out-of-frame, drop-in Ribo-seq density at the stop codon and so on) to rank smORFs for high-confidence of translation. Pooling data in these studies provided increased evidence to define the boundaries of smORFs and allowed testing for their translation more stringently. Specifically, for the two examples described above in Figure 5, by pooling data the codon coverage increased from ~ 20% to ~ 80%. Using the merged data, the difference between a truly translated and false positive is clearer, such as SEHBP (a functionally validated, stable peptide [44], Figure 5D) which is known to be translated has a clear translation signature opposed to a false-positive smORF shown in Figure 5E does not have a translation signature even after merging data. In order to demonstrate the global impact of sequencing depth on ORF detection, we downsampled published pooled Ribo-seq data [31] to 6 million and up to 1.3 billion inferred P-sites. We then quantified the detection rate of known ORFs and predicted smORFs from the same study across a range of total inferred P-sites (using a codons-in-frame value of 70% as a detection threshold) (see Figure 6A–D). This demonstrates that with ~ 6 million inferred P-sites, the vast majority of ORFs and smORFs have poor codon coverage (Figure 6A) and as a result only ~ 3% (or 431) of expressed known ORFs and ~ 1% (or 103) of smORFs would be detected (Figure 6C,D). Pooling samples increase codon coverage and thus enable us to have stricter quality control by testing each codon within the smORF for translation. Thus, allowing higher resolution to identify the translation as well as the accurate start of the smORF. As smORF reference sets are in early stages, similar to what has been done historically for gene models, smORFs initial set can be defined using pooled data which can be further refined in future versions.

## Current reference sets use different data, methods, and assumptions

Previous cataloguing efforts have allowed researchers access to reference sets of SEPs that can be further tested in a given system of interest (see Table 1). OpenProt [25] and the uORFdb (version 2) [26,27] identify all possible ORFs and uORFs, respectively, using a 3-frame translation of the transcriptome while grouping similar ORFs. OpenProt further annotated several pieces of evidence such as protein conservation based on sequence homology, expression based on mass-spectrometry-based proteomics and translation based on Ribo-seq. Specifically, they used published Ribo-seq data to detect smORFs using PRICE [53] and found only 5,696 of the 461,462 reported on OpenProt with evidence of translation. Not all ORF sequences may be translated and have just occurred by chance, thus such comprehensive approaches have the risk of increased type I error (more false positives). SmProt (version 1) [68], uORFdb [26,27], MetamORF [28] curate smORFs detected using Ribo-seq from the literature and collate them on a database. Such “union sets’’ have allowed an overview of smORFs found in any published study but led to millions of smORFs catalogued together that were identified from dataset with varying quality and 3-nt periodicity leading to many false positives. In order to reduce the false positives, a study limited their data-source to only seven high-quality Ribo-seq dataset and combined the set of smORFs published in these individual studies [29]. This study presents the Phase I smORFs as the ones found in at least two studies, or an ‘intersection set’ as high-confidence smORFs based on repeated identification. Although replicability can show repeated evidence of a Ribo-seq signal, it does not indicate that the ones that are not replicated are not translated. Both the union set and intersection set approaches, combined smORFs identified using varying data quality, different computational methods and detection criterion leading to discrepancies in what is considered as a translated smORF.

**Table 1.** Comparison of data, methods and presets used to catalogue human smORFs.

	Uses Ribo-seq to detect smORFs	No. of Ribo-seq samples used	Cell-types/tissues	Uniform Re-processing of Ribo-seq data	Individual/merged data used	Method to call smORFs	Method to select most probable isoform	Start-codon	Length threshold (amino acids)	No. of smORFs reported	uORFs: nuORFs: dORFs reported
sorfs.org	Y	34 dataset	Various	Y	Only replicates merged	Unified	N	AUG and near-cognate	10	555,927	-
SmPROT v2	Y	96 samples	Various	Y	Only replicates merged	Unified	Using the – frame best score	AUG and near-cognate	5	327,995	NA
OpenPROT	N	NA	NA	N	NA	NA	N	AUG	30	461,462	NA
MetaMORF	Y	-	Various	N	Various	Various	N	Various	No	664,771	-
uORFdb	N	NA	Various	N	NA	NA	N	AUG and near-cognate	No	>2.4 million	Only uORFs
Mudge et al.	Y	139samples	Various	N	Various	Various	Longest ORF	AUG	16	7,264	3771: 2208: 565
Chothani et al.	Y	187 samples	Various	Y	Merged	Unified	Longest ORF with best uniformity score	AUG and near-cognate	No	7,767	5280: 1652: 802

In order to have a consistent definition of smORF translation, several studies detect smORFs in a unified manner by uniformly processing data and applying common tools and presets [30,32,69]. SmProt [30] (version 2) re-processes 96 human Ribo-seq samples and detects translated smORFs using RiboTISH [59] on individual samples. Sorfs.org uses 34 human Ribo-seq datasets and uses an in-built pipeline to detect smORFs present in individual samples [32]. To account for low-depth in datasets, sorfs.org employs a lenient threshold of 10% in-frame coverage and tests for recurrence of smORFs in multiple datasets. These studies have allowed standardized identification of smORFs but due to sparseness in Ribo-seq samples makes it difficult to distinguish between actual translation and noise as described previously. In humans, a recent study merged high-quality Ribo-seq data to mitigate the sparseness in the data [31] and detected smORFs with a unified pipeline. Pooled data allowed higher codon coverage, thus allowing to test each codon within the smORF and stricter QC, only selecting smORFs which have a high 3-nt periodicity (>75%), percentage of codons-in-frame (>71%) and drop-off score (>92%). Uniform processing and standard definition allows for a more straightforward interpretation of catalogued smORFs as opposed to combining smORF lists from different dataset which were called using a variety of data, tools and assumptions.

## What have current smORF sets told us about nuORFs, uORFs, and dORFs?

The reference sets for smORFs, while still evolving, have already enhanced our knowledge on their global properties such as their overall abundance, expression, start-site usage and evolutionary conservation. In humans, uORFs (upstream ORFs) have been found to be most abundant followed by nuORFs (novel unannotated ORFs), and dORFs (downstream ORFs) being the fewest in number across the human genome [29,31]. Human smORFs have also been shown to bemore recently evolved, especially the ones in lncRNAs [19,31]. The generation of a reference set has also allowed quantification of expression levels and translation efficiency for each smORF and several studies have found that uORFs are generally comparable to known ORFs in their translation levels and TE whereas dORFs have been found to have lower levels as compared to known ORFs [31,49]. NuORFs have been found to have low translation levels but the translation-efficiency for nuORFs is nearly comparable to known ORFs [49]. With regard to start-site usage, generally more than half of the translated smORFs have been found to use non-AUG start-codons [9,61,62]. Specifically, uORFs are more enriched for non-canonical start-codons compared to other ORFs [9,49]. Translated uORFs have been found more often in genes encoding transcription factors [31], oncogenes and cellular receptors [70]. These global properties have allowed us to view smORFs and known ORFs together to be able to understand the similarities and differences between them, their potential functions and evolutionary history.

Apart from global properties of smORFs, several different possibilities of functions of their translation have been described. NuORFs have been generally linked to generate functional proteins that are important in humans such as for heart development [71], muscle formation [72–74], regulating calcium uptake in muscle [75,76] and play important roles in the mitochondria [77–79]. For smORFs in the untranslated regions of known protein-coding ORFs there are various alternative fates [80]. UORFs have been frequently shown to repress the known ORF on its transcript and few global studies show lower readout of protein levels for known ORFs containing uORFs using proteomics readout [70,81] and translation-efficiency [49,58]. Although, several negatively regulated uORF-mORF pairs exist, translation of uORFs has also been shown to positively affect the translation of the main ORF [4,82,82,83] and protect the translation of the known ORF under stress [84]. Recently, several human genome-wide studies have highlighted that although there do exist several uORF-mORF pairs that are negatively regulated, the predominant trend shows uORF and mORF being regulated in the same direction. This has been shown independently by various studies, such as for cell-identity of fibroblasts, endothelial cells, kidney, brain and heart tissues [31] and in disease conditions such as fibrosis [85] and in glioblastoma [5] and dilated cardiomyopathy patients [10]. Another study found deleting start-codon for peptide-forming uORFs only minimally increased the expression of the main CDS indicating non repressive function of uORFs [6]. uORFs are also increasingly being shown to encode peptides with important functions in disease [5], and form complexes or directly inhibit other proteins [6,7]. Apart from uORFs, dORFs, which are found on the 3’UTR of known ORFs, have been shown to enhance translation of the main ORFs and the number of dORFs rather than the length is shown to further enhance this effect [86]. A study also showed a dORF encoded a protein that is a cancer antigen [87]. While these studies show evidence for the possible function of smORFs and their encoded peptides, with 1000s of smORFs detected in Ribo-seq with translation signatures identical to known proteins, more studies are needed to understand the roles of nuORFs, uORFs and dORFs.

## Road ahead and challenges

There is a growing concern in the scientific community that the current reference set of long ORFs may have overlooked a significant number of smORFs. This has led to a community call for the development of a translated smORF reference set that can be integrated into existing annotations [29]. Currently available Ribo-seq data have shallow-depth and are sparse in nature and thus to detect smORFs accurately it requires pooling of data, as has been presented for human smORFs [31]. This study uniformly processed, analysed and pooled high-quality data to obtain >80% codon coverage on the human translatome and thus was able to detect a reference set of smORFs that have undergone a stricter quality control by testing each codon for translation. Moving forward, we recommend pooling existing high-quality data for a given species to account for the data-sparseness and uniformly identify smORFs to obtain a reference set. All subsequent newly generated Ribo-seq data should then be added to the original data release to re-analyse and identify smORFs further improving the annotation. To ensure stability across versions, two primary areas need to be considered. First, the approach to updating and sharing revisions and second, the method for ranking smORFs to provide a confidence level. Learning from the experience of incorporating known ORFs into the genebuild can help in developing a smORF reference set. Ensembl is updated every 3 months and significant updates, such as the most recent genome build was updated after 5 years. Similarly, smORF reference set updates should be released every few years with new Ribo-seq data pooled with previous data builds to identify and update the smORF reference set. Criteria should be established to add or remove smORFs. Instead of removing smORFs with slightly reduced scores in newer versions, they could be assigned a confidence level based on consistent or improved uniformity and periodicity in reference set updates. A translation support level (TrSL) can be assigned to smORFs similar to transcript support level (TSL) scores provided by Ensembl for transcripts. With technological advances from cDNA sequencing to RNA-seq with longer sequencing reads, revisions of gene models have now become fairly consistent. Future revisions of smORF reference sets will also need to be dynamic and aim to follow the same trajectory, and thus, be updated with not only new data but also incorporate advances in Ribo-seq protocols improving data resolution and quality.

A reference set of translated smORFs can be a powerful tool for discoveries of new proteins or regulatory control elements. Previously conducted studies to understand known ORFs can be used as blueprints to discover smORF biology. Here, we provide a few examples of applications in each layer of protein production, i.e. DNA, RNA and protein. At the DNA level, a study showed uORF start-creating and stop-disrupting mutations are under strong negative selection [88]. smORFs can be tested for presence of GWAS and eQTLs and have been reviewed for cardiovascular disorders recently [89]. At the RNA level, the smORFs can be used for differential expression analysis using RNA-sequencing and Ribo-seq to understand their role in a given biological system [12]. Generation of a reference set incorporated along with the known ORFs would allow researchers to use publicly available RNAseq data to infer which smORFs are differentially regulated in a given disease or perturbation of interest without having to perform Ribo-seq and call smORFs in every new system of study. For those interested to investigate lncRNA coding potential, several sequence- and evolutionary conservation-based tools [90,91], and more recently deep learning models, have been developed [92,93] to identify cryptic ORFs using *in silico* prediction. As has recently become evident, lncRNAs tend to encode young proteins [19] and thus traditional ORF prediction methods which rely on length-biased and evolutionary conservation-biased methods would not be able to discern coding potential efficiently [46]. Instead, Ribo-seq provides experimental evidence for translation within lncRNAs and thus smORF reference sets can be used as a direct-measure of lncRNA coding potential. Tools such as ‘Is it a smORF?’ available in http://smorfs.ddnetbio.com/ can be used to identify lncRNAs encoding high-confidence translated smORFs using ribo-seq evidence. Lastly, protein-level studies can help us delineate whether a smORF makes a stable peptide or is degraded. Thus, several studies have verified the evidence of their presence in mass spectrometry data [11,25,31,32,52,94–96], but due to technical limitations [97,98] for detecting short peptide sequences accurately, best practices for methods to detect smORFs in-vivo are still developing. CRISPR-based screening strategies have also been deployed to identify smORFs essential for cellular growth [6,99] and cancer cell survival [99]. Depending on the number of smORFs that can be directly used for testing, the reference set may need to be filtered to obtain a more feasible number. The activity and role of smORFs can be better understood with such studies and thus have the potential to uncover new insights into cellular processes as well as disease mechanisms. As our understanding of smORFs grows, they can be incorporated into widely used databases such as Uniprot, GENCODE and Ensembl similar to the approach taken with known protein-coding ORFs, further expanding our knowledge of the translatome and proteome.

## Supplementary Material

Supplemental Material

## Data Availability

Data sharing is not applicable to this article as no new data were generated in this study.
