# Supplementary material for: Discovering microproteins: making the most of ribosome profiling data
Source: RNA Biol. 2023 Nov 27;20(1):943–54. doi: 10.1080/15476286.2023.2279845 (PMC10730196; doi:10.1080/15476286.2023.2279845)
Supplement: Supplemental Material [file KRNB_A_2279845_SM0709.docx]

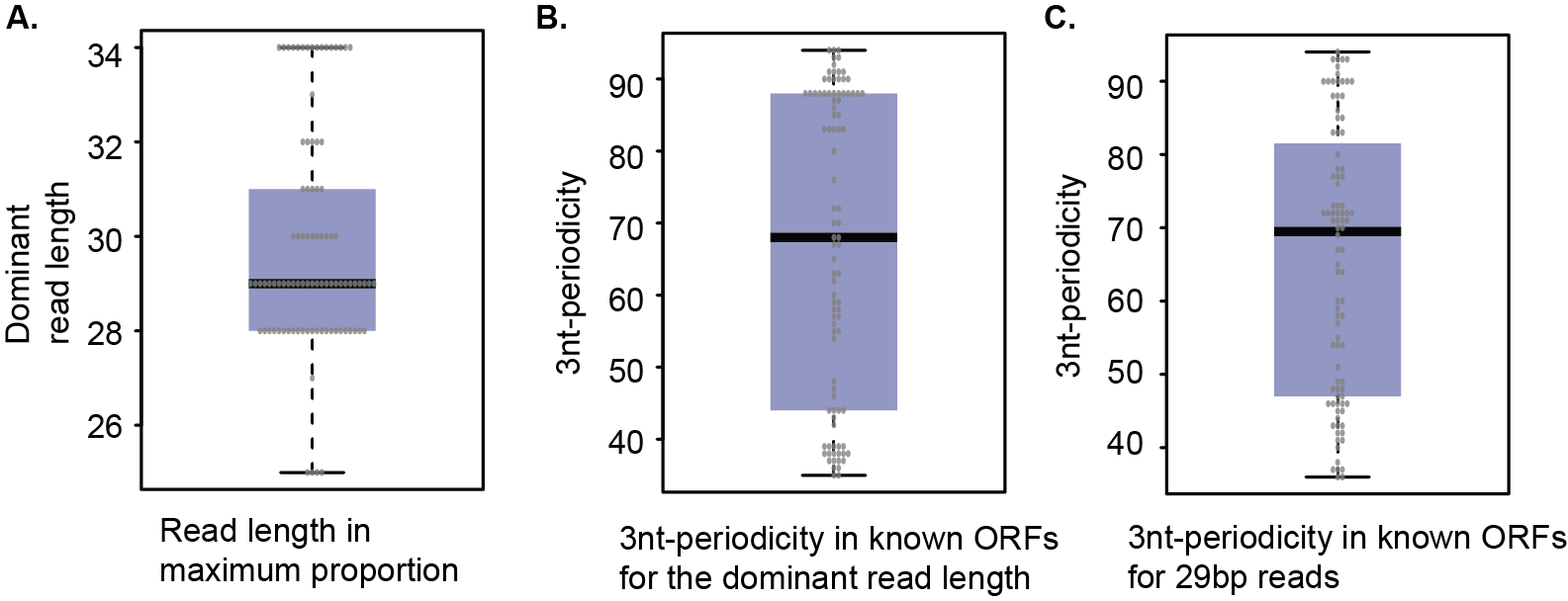


**Supplementary Figure 1: A.** Box plot showing the read length of maximum number of reads (dominant read length) in each sample.**B.** Box plot showing the 3nt-periodicity in known ORFs using the respective dominant read length for the given sample. **C.**Box plot showing 3nt-periodicity in known ORFs using 29 bp read lengths within a given sample. Fifteen human datasets (92 samples) were randomly selected from RPFdb and the read lengths and periodicity values were obtained from the quality figure files on the database.
